# Supplementary material for: Raman spectroscopy on blood serum samples of patients with end-stage liver disease
Source: PLoS One. 2021 Sep 7;16(9):e0256045. doi: 10.1371/journal.pone.0256045 (PMC8423274; doi:10.1371/journal.pone.0256045)
Supplement: S1 Table — (PDF) [file pone.0256045.s001.pdf]

Raman spectroscopy on blood serum samples of patients with end-stage liver disease - Supplementary Material

René Staritzbichler[1,‡,*] Pascal Hunold[1,2], Irina Estrela-Lopis[1], Peter W. Hildebrand[1], Berend Isermann[2], Thorsten Kaiser[2,‡,*]

1 Institute for Medical Physics and Biophysics, Leipzig University, Leipzig, Germany
2 Institute of Laboratory Medicine, Clinical Chemistry and Molecular Diagnostics, University Hospital Leipzig, Leipzig, Germany

‡: These authors contributed equally to this work.
* rene.staritzbichler@medizin.uni-leipzig.de and thorsten.kaiser@medizin.uni-leipzig.de.

Number of sample scans	30
Collection length	104.00 sec
Number of background scans	512
Raman laser wavenumber	12817.90 cm^{-1}
Number of data points	3371
Min/Max/Delta wavenumber	50.5/3300/0.96 cm^{-1}

Table S1: Used settings of the DXR SmartRaman.
