# Supplementary figures and images for: Raman spectroscopy on blood serum samples of patients with end-stage liver disease
Source: PLoS One. 2021 Sep 7;16(9):e0256045. doi: 10.1371/journal.pone.0256045 (PMC8423274; doi:10.1371/journal.pone.0256045)

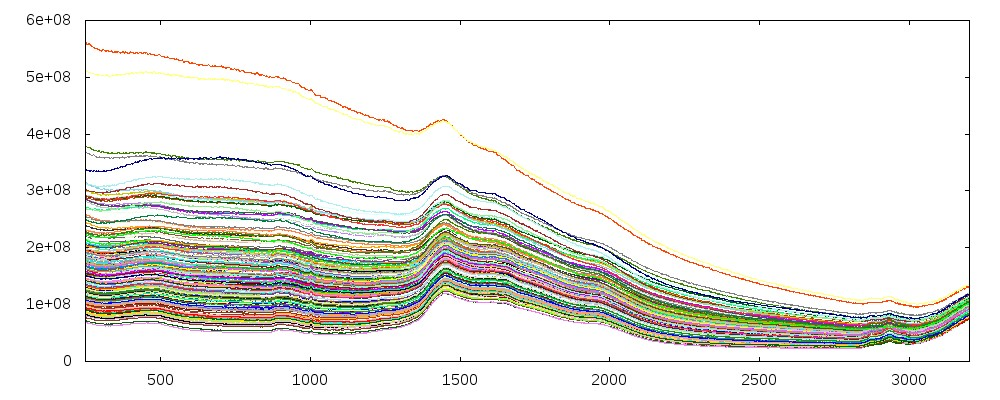

Supplement: S1 Fig — In red is an arbitrary spectral vector S→i illustrated, for a wavenumber of ∼2000 cm−1. (TIF) [file pone.0256045.s002.tif]

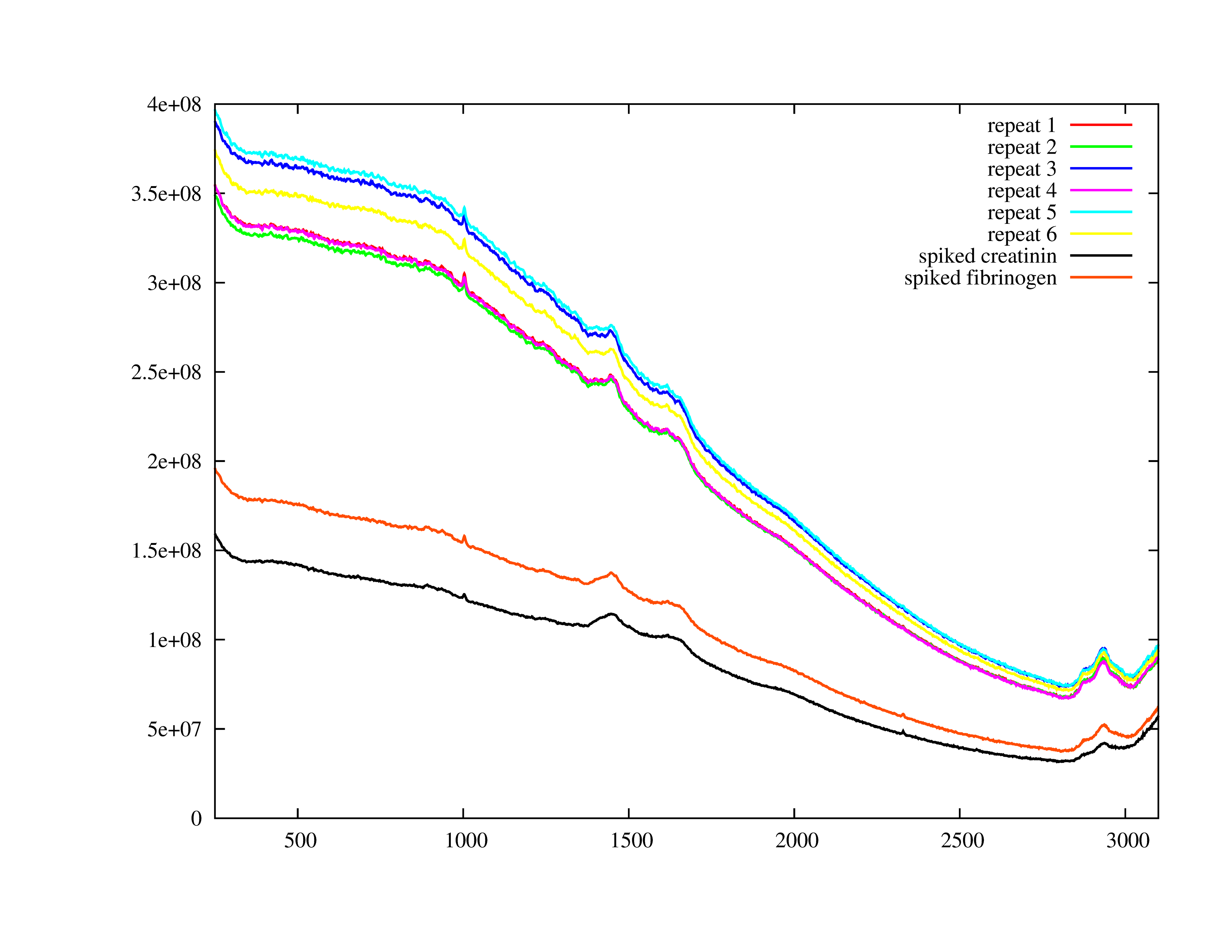

Supplement: S2 Fig — Each spectrum shows repeated measurements of a single patient, respectively. The first six curves are independent spectra from the same sample. The last two spectra are taken using the same sample, spiked with 1:1 Creatinine and Fibrinogen solution (final concentrations: creatinine: 5mmol/l, fibrinogen 10g/l). The enrichment was performed to detect specific peaks caused by the additives. However, no specific peak could be assigned from visual inspection. Exposure time: 172s. (TIF) [file pone.0256045.s003.tif]
